# Fixed airways obstruction among patients with severe asthma: findings from the Singapore General Hospital-Severe Asthma Phenotype Study

**DOI:** 10.1186/1471-2466-14-191

**Published:** 2014-12-03

**Authors:** Anthony Chau Ang Yii, Gan Liang Tan, Keng Leong Tan, Therese Sophie Lapperre, Mariko Siyue Koh

**Affiliations:** Department of Respiratory and Critical Care Medicine, Singapore General Hospital, 20 College Road, Singapore, 169856 Singapore

**Keywords:** Fixed airways obstruction, Severe asthma, Smoking, Air trapping

## Abstract

**Background:**

A subset of severe asthma patients has fixed airways obstruction, which is characterized by incomplete reversibility to bronchodilator challenge. We aimed to elucidate the factors associated with fixed airways obstruction in a cohort of patients with severe asthma in Singapore.

**Methods:**

245 patients from the Singapore General Hospital-Severe Asthma Phenotype Study (SGH-SAPS) were screened. These patients fulfilled World Health Organization criteria for "treatment-resistant severe asthma" and were all on combination of high-dose inhaled corticosteroids and long-acting beta2 agonists. 76 patients had pre- and postbronchodilator lung function tests and were selected for analysis. They were divided into two groups based on postbronchodilator (Post BD) forced expiratory volume in one second, PostBDFEV_1_ % predicted: ≥70% (Non-Fixed Obs) and < 70% (Fixed Obs). We compared clinical and demographic parameters between the two groups.

**Results:**

Patients in the Fixed Obs group were more frequently past or current smokers and had a higher pack-year smoking history. Overall, pack-year smoking history had a modest negative correlation with PostBDFEV_1_ % predicted. Atopy, allergen sensitization (type and numbers), comorbidities, symptoms, health care utilization and medication use did not differ between the two groups. The prebronchodilator FEV_1_ % predicted, FEV_1_/FVC and FVC % predicted were significantly lower in the Fixed Obs group. In addition, prebronchodilator FVC % predicted accounted for more variability than FEV_1_/FVC in predicting PostBDFEV_1_% predicted.

**Conclusion:**

Smoking is associated with fixed airways obstruction in patients with treatment-resistant severe asthma in Singapore. Furthermore, our results suggest that both small and large airways obstruction contribute independently to fixed airways obstruction in severe asthma.

## Background

Asthma affects an estimated 315 million adults worldwide [1] and incurs substantial health care and socioeconomic costs [2]. Only 5% of all asthmatics have severe asthma, but they disproportionately account for most of the morbidity, mortality and health care burden across all asthma patients [3].

Even among those with severe disease, asthma is heterogeneous and consists of multiple phenotypes [4]. The fixed airways obstruction phenotype occurs in a small proportion of patients who experience irreversible airways obstruction despite inhaled corticosteroids or bronchodilators [5, 6]. Fixed airways obstruction in asthma is associated with more frequent exacerbations [7], increased asthma-related mortality [8] and overall mortality [9]. The causes of fixed obstruction in asthma are unknown but may be related to the presence of airway wall remodeling, which is characterized by increased airway smooth muscle mass and airway wall fibrosis [10].

Studies in American [11], European [12–15], Canadian [16] and East Asian [17, 18] cohorts have identified diverse but sometimes conflicting risk factors for fixed airways obstruction in asthma, including male sex, older age, longer duration of illness, smoking, eosinophilic airway inflammation, atopy and increased airway hyper-responsiveness. These inconsistent results could reflect differences in study methodologies, or the influence of gene-environment factors. We sought to elucidate the factors associated with fixed airways obstruction in Singaporean patients with severe asthma. To our knowledge, this is the first report to characterize fixed airways obstruction among asthmatics in a Southeast Asian cohort.

## Methods

### Patient characteristics

We interrogated the Singapore General Hospital-Severe Asthma Phenotype Study (SGH-SAPS) database, which consisted of 245 patients with severe asthma who presented to the Allergy or Respiratory clinics at Singapore General Hospital between 1 January 2011 and 31 December 2012. The diagnosis of asthma was made on the basis of history of episodic wheeze and dyspnea, clinical examination and supported by spirometry where indicated (reversibility of forced expiratory volume in one second (FEV_1_) of > 12% and 200 ml, or demonstration of bronchial hyper-responsiveness with a positive methacholine challenge test, or FEV_1_ variability of > 15%). Diagnosis of asthma was made by experienced clinicians in the field of respiratory and allergy in our hospital. Severe asthma was defined according to the World Health Organization classification of "treatment-resistant severe asthma" [19], that is, patients who require a combination of high-dose inhaled corticosteroids and long-acting beta agonists. Patients were considered to have treatment-resistant severe asthma based on the above criteria and following a period of treatment optimization, assessment of adherence, and identification and treatment of other comorbidities. The institution’s ethics committee approved the study and waived the requirement for informed consent.

**Figure 1:**
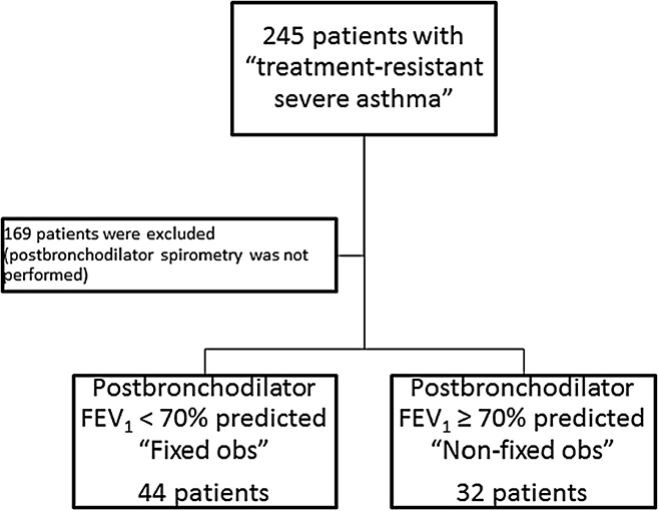
**Study participants.**

For the present analysis, we included 76 of the 245 patients (Figure 1) for whom postbronchodilator spirometry data were available and divided them into two groups based on postbronchodilator FEV_1_ % predicted: < 70% ("Fixed Obs", n = 44) and ≥ 70% ("Non-fixed Obs", n = 32).

### Design

This was a retrospective cross-sectional study.

### Measurements and investigations

Asthma symptoms were evaluated using the Asthma Control Test [20]. We assessed for the presence of asthma-related comorbidities of interest (allergic rhinitis, eczema, reflux, obstructive sleep apnea, anxiety, depression, aspirin sensitivity and vocal cord dysfunction) by obtaining history and reviewing medical records. The number of admissions, emergency visits, steroid bursts and medications over a two year period (2011–2012) were determined by questioning patients and cross-checking the national network of electronic health records, which contains discharge summaries, investigation results, emergency department records and electronic prescriptions from all public hospitals in Singapore.

Patient medications were coded according to an ordinal scale as follows: 1 = inhaled corticosteroid only, 2 = combination inhaled corticosteroid and beta-agonist (Combi), 3 = Combi + theophylline, 4 = Combi + leukotriene receptor antagonist (LTRA), 5 = Combi + Theophylline +  LTRA, 6 = Long term steroids, 7 = Omalizumab, 8 = Anticholinergic, 9 = All of the above.

Spirometry was performed according to American Thoracic Society/European Respiratory Society guidelines [21], using a Medgraphics, USA spirometer. Predicted values were obtained from *Morris et al.* [22] and an adjustment factor of 0.94 was applied for FEV_1_ and forced vital capacity (FVC) as recommended for Asian patients [23]. Reversibility in FEV_1_ was measured 10 to 15 minutes after administration of 400 μg inhaled salbutamol with a spacer, and was expressed as change in percentage predicted and absolute change in mls.

Skin prick tests were also retrospectively assessed and were performed with a panel (Stallergenes, France) which included the following extracts: dust mites (*Bloomia tropicalis, Dermatophagoides pteronyssinus* and *farinae*), dog and cat danders, feathers, cockroach, and molds (*Aspergillus fumigatus*), a negative control (glycerine) and positive control (histamine, 10 mg/ml). A positive response was defined as any wheal with a diameter 3 mm greater than the negative control, 15 minutes after application.

### Statistical analysis

Data were expressed either as mean ± standard deviation (SD) for normally distributed continuous variables or alternatively as median (interquartile range) for ordinal variables and for non-normally distributed continuous variables. Comparisons between groups were performed using the Student’s t-test for normally distributed continuous variables, the Mann–Whitney U test for ordinal and for non-normally distributed continuous variables, or the Chi-squared test for proportional data, as appropriate. Correlations were investigated using either the Spearman or Pearson tests. Hierarchical multiple linear regression was used to assess the relationship between prebronchodilator FEV_1_ and FEV_1_/FVC with PostBDFEV_1_. All variables found to be significant correlated with postbronchodilator FEV_1_ were evaluated for collinearity using a cut-off of tolerance < 0.2 or variable inflation factor > 5. Only predictor variables found to be non-collinear were included in multiple linear regression analysis employing the Automatic Linear Modelling module in SPSS version 20. Statistical procedures were carried out with IBM SPSS version 20. P values of less than 0.05 were considered significant.

## Results

### Patient demographics

The characteristics of the SGH-SAPS cohort are reported in Table 1. The distribution of ethnicity among the cohort as compared to the general population in Singapore was as follows: Chinese (66.5% vs. 76.8%), Malay (12.2% vs. 13.9%), Indian (15.1% vs. 7.9%), others (6.1% vs. 1.4%). Overall, 44 patients (18%) out of our cohort of 245 severe asthmatics had evidence of fixed airways obstruction, according to the definition of postbronchodilator FEV_1_ < 70% predicted. Among the 76 patients with bronchodilator testing, the mean age was 54.4 ± 18.2 years, the mean age of onset was 35.5 ± 22.6 years. Age, age of onset, duration of asthma, sex and body mass index were not different between the Fixed Obs and Non-Fixed Obs groups (Table 2). The mean and 95% confidence interval (CI) for age and age of onset for the Fixed Obs group were 52.3 yrs ± 18.1 (95% CI: 46.8-57.8 yrs) and 32.8 ± 22.9 yrs (95% CI: 25.8-39.8 yrs), respectively.

**Table 1:** **Baseline characteristics of the Singapore General Hospital-Severe Asthma Phenotype Study cohort**

	Overall cohort	Study participants	P-value (participants vs. non-participants)
Patients (n)	245	76	
Age (years)	53.1 ± 19.0	54.4 ± 18.2	NS
Age of asthma onset (years)	32.2 ± 21.5	35.5 ± 22.6	NS
Duration of asthma (years)	20.9 ± 16.7	18.9 ± 15.9	NS
Gender (% Males)	47.3	50	NS
Ethnicity (%)			NS
Chinese	66.5	65.8	
Malay	12.2	10.5	
Indian	15.1	18.4	
Others	6.1	5.3	
Current or past smokers (%)	21.6	28.9	NS
Smoking history (pack-years)	0 (0–0) Range 0-64	0 (0–1) Range 0-64	NS
Prebronchodilator FEV_1_ % (predicted)	72.2 ± 22.5	54.9 ± 17.2	<0.001

Data is presented as mean ± SD, proportion or median (interquartile range). FEV_1_ = forced expiratory volume in one second, NS = not significant.

**Table 2:** **Comparisons between subjects with fixed and non-fixed airways obstruction**

Characteristic	< 70% ("Fixed Obs)	≥ 70% ("Non-Fixed Obs")	p-value
Patients (n)	44	32	
Age (years)	52.3 ± 18.1	57.4 ± 18.2	NS
Age of asthma onset (years)	32.8 ± 22.9	39.3 ± 21.9	NS
Before 12 years old (%)	34.1	18.8	NS
Before 18 years old (%)	36.4	18.8	NS
Before 40 years old (%)	50.0	50.0	NS
Duration of asthma (years)	19.5 ± 17.6	18.1 ± 13.5	NS
Gender (% Males)	56.8	40.6	NS
Body mass index (kg/m^2^)	25.2 ± 7.3	25.0 ± 5.5	NS
**Serum eosinophils**			
No. of patients (n)	43	31	
Absolute eosinophil count (× 10^9^/L)	0.49 ± 0.73	0.46 ± 0.43	NS
Eosinophil count ≥ 0.4 × 10^9^/L (%)	37.2	51.6	NS
**Smoking**			
Current or past smokers (%)	38.6	15.6	0.029
Smoking history (pack-years)	0 (0–10)	0 (0–0)	0.019
	Range: 0-64	Range: 0-30	
**Comorbidities**			
Allergic Rhinitis (%)	45.5	53.1	NS
Eczema (%)	100	100	NS
Gastroesophageal reflux disease (%)	15.9	12.5	NS
Obstructive sleep apnea (%)	6.8	0	NS
Anxiety (%)	6.8	0	NS
Depression (%)	2.3	3.1	NS
Aspirin sensitivity (%)	0.0	3.1	NS
Vocal cord dysfunction (%)	2.3	0	NS
**Skin prick tests**			
No. of patients (n)	14	14	
Positive results on skin prick test to:			
*Blomia tropicalis* (%)	78.6	92.9	NS
*Dermatophagoides pteronyssinus* (%)	85.7	85.7	NS
*Dermatophagoides farinae* (%)	85.7	85.7	NS
Dog (%)	64.3	50.0	NS
Cat (%)	50.0	50.0	NS
Feathers (%)	14.3	21.4	NS
Cockroach (%)	28.6	14.3	NS
*Aspergillus* (%)	14.3	7.1	NS
No. of allergens which test positive	5 (2.75-6)	4 (3–5.25)	NS
**Symptoms, health care use, medications**			
Asthma Control Test score	20 (16–22)	20 (19–24)	NS
Admissions in the past 2 years	0 (0–0) (n = 42)	0 (0–2) (n = 31)	NS
% admitted in past 2 years	14.3	32.3	NS
Emergency visits in the past 2 years	0 (0–2) (n = 43)	1 (0–2) (n = 31)	NS
% with emergency visits in past 2 years	48.8	58.1	NS
No. of steroid bursts in the past year	1 (0–2)	1 (0–2)	NS
% with steroid bursts in the past year	61.4	71.9	NS
History of near-fatal asthma (%)	4.5	9.4	NS
Medication regimen	2 (2–4)	2 (2–4)	NS
**Lung function**			
PostBDFEV_1_ (% predicted)			
Range	32-69	71-123	NA
Prebronchodilator FEV_1_ (% predicted)	45.1 ± 10.0	68.4 ± 15.9	<0.001
Prebronchodilator FVC (% predicted)	56.8 ± 16.8	74.6 ± 15.1	<0.001
Prebronchodilator FEV_1_/FVC (%)	61.3 ± 14.1	70.9 ± 12.0	0.003
Percentage reversibility in FEV_1_ (%)	26.8 ± 24.2	25.9 ± 18.6	NS
Reversibility in FEV_1_ (ml)	283 ± 222	360 ± 242	NS

Data is presented as mean ± SD, proportion or median (interquartile range).

FEV_1_ = forced expiratory volume in one second, FVC = forced vital capacity, NS = not significant, NA = not applicable. Medication regimen is expressed as an ordinal scale as follows: 1 = inhaled corticosteroid only, 2 = combination inhaled corticosteroid and beta-agonist (Combi), 3 = Combi + theophylline, 4 = Combi + receptor antagonist (LTRA), 5 = Combi + Theophylline + LTRA, 6 = Long term steroids, 7 = Omalizumab, 8 = Anticholinergic, 9 = All of the above.

### Smoking

**Figure 2:**
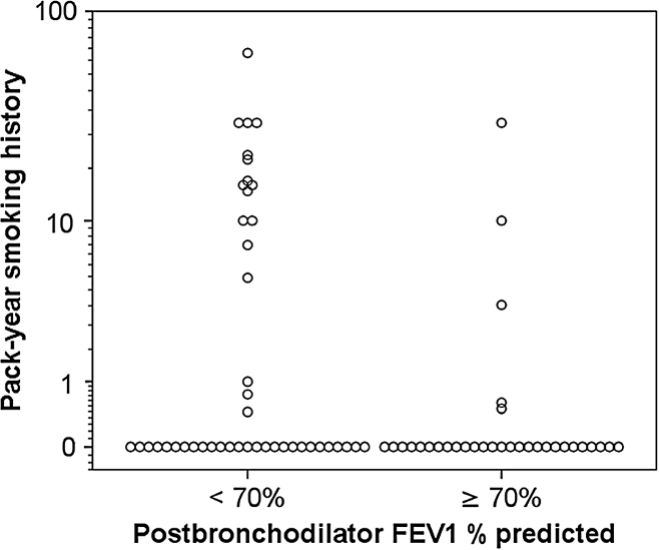
**Pack-year smoking history of subjects according to postbronchodilator FEV**_**1**_ **% predicted.** Patients with postbronchodilator FEV_1_ % < 70% had a higher pack-year smoking than patients with postbronchodilator FEV1 % ≥ 70% (Mann–Whitney, p = 0.022).

**Figure 3:**
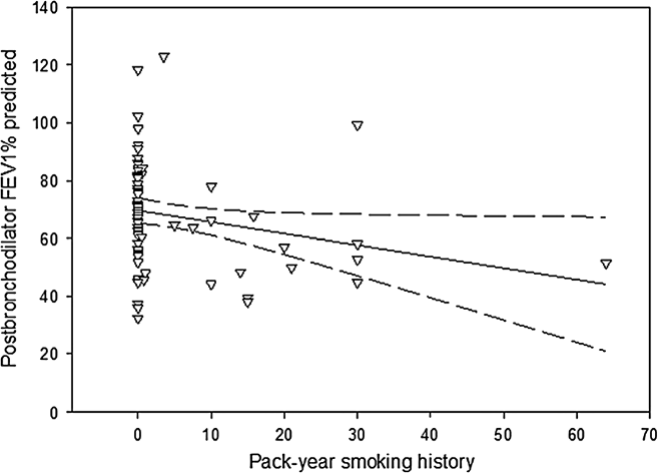
**The relationship between postbronchodilator forced expiratory volume in one second % predicted versus pack-year smoking history.** Solid and dashed lines denote the fitted linear regression curve and 95% confidence interval, respectively.

Table 2 and Figure 2 show the smoking history of subjects according to PostBDFEV_1_ % predicted. The proportion of current or past smokers was significantly higher for the Fixed Obs group compared to the Non-Fixed Obs group (p = 0.034). The former group also had a significantly higher pack-year history than the latter (p = 0.022). In addition, there was a significant negative correlation between pack-year smoking history and PostBDFEV_1_ % predicted (p = 0.004, r_s_ = -0.306) with pack-year smoking history accounting for approximately 9% of variability in PostBDFEV_1_ % predicted (Figure 3).

### Atopy, allergy tests and comorbidities

The prevalence of atopic diseases (allergic rhinitis, aspirin sensitivity, eczema), allergen sensitization based on skin prick tests, prevalence of other asthma-related comorbidities such as gastroesophageal reflux, obstructive sleep apnea, vocal cord dysfunction, anxiety and depression were not different between the Fixed Obs and Non-Fixed Obs groups (Table 2).

### Symptoms, psychological dysfunction, health care utilization and medications

Symptom control as measured by the Asthma Control Test was not different between groups. In addition, there were no between-group differences in terms of emergency visits, hospital admissions, near-fatal attacks, number of short courses of oral steroids in the past year, and asthma medication regimen.

### Lung function

The prebronchodilator FEV_1_ % predicted, FEV_1_/FVC and FVC % predicted were significantly lower in the Fixed Obs group compared to the Non-Fixed Obs group.

### Correlational analysis

Bivariate analyses of the entire study sample (n = 76) demonstrated that postbronchodilator FEV_1_ % predicted was significantly correlated with the following continuous or ordinal variables (Table 3): pack-year smoking history, prebronchodilator FEV_1_, FVC, and FEV_1_/FVC, and admissions in the past year. Collinearity diagnostics revealed that among all the significantly correlated variables, prebronchodilator FEV_1_ had a high degree of multicollinearity (tolerance = 0.175, variable inflation factor = 5.717), hence it was removed from the final multiple linear regression model. Multivariate regression incorporating all other variables found to be significantly correlated with postbronchodilator FEV_1_ % predicted (Table 4) showed that the following variables were significantly and independently associated with postbronchodilator FEV_1_, in order of decreasing importance: prebronchodilator FVC % predicted, prebronchodilator FEV_1_/FVC and smoking.

**Table 3:** **Variables correlated with postbronchodilator forced expiratory volume in one second % predicted**

Variable	Correlation coefficient	p-value
Age (years)	0.203^#^	0.08
Age of onset (years)	0.200^#^	0.09
Duration of asthma (years)	-0.056^#^	0.63
Body mass index (kg/m^2^)	0.029^#^	0.80
Serum eosinophils (× 10^9^/L)	-0.092^#^	0.43
Pack-year smoking history	-0.306*	0.007
Asthma control test score	0.143*	0.22
No. of emergency visits in past two years	0.102*	0.39
Steroid bursts in the past year	0.102*	0.38
Medication regimen	0.014*	0.90
Admissions in the past year	0.278*	0.015
Prebronchodilator FEV_1_ (% predicted)	0.882^#^	<0.001
Prebronchodilator FVC (% predicted)	0.650^#^	<0.001
Prebronchodilator FEV_1_/FVC (%)	0.425^#^	<0.001
Reversibility (%)	0.012^#^	0.92
Reversibility (ml)	0.163^#^	0.16

Medication regimen is expressed as an ordinal scale as follows: 1 = inhaled corticosteroid only, 2 = combination inhaled corticosteroid and beta-agonist (Combi), 3 = Combi + theophylline, 4 = Combi + leukotriene receptor antagonist (LTRA), 5 = Combi + Theophylline + LTRA, 6 = Long term steroids, 7 = Omalizumab, 8 = Anticholinergic, 9 = All of the above.

^#^Pearson coefficients, *Spearmann coefficients. FEV_1_ = forced expiratory volume in one second, FVC = forced vital capacity.

**Table 4:** **Multiple linear regression of factors correlated with postbronchodilator FEV**_**1**_ **% predicted**

Factor	Coefficient	p-value	Importance
Prebronchodilator FVC (% predicted)	0.709	<0.001	0.666
Prebronchodilator (FEV_1_/FVC %)	0.606	<0.001	0.294
Pack-year smoking history	-0.293	0.049	0.024
Admissions in the past year	-4.921	NS	0.016

### Relative importance of prebronchodilator FEV_1_/FVC and FVC % predicted in predicting bronchodilator reversibility

**Figure 4:**
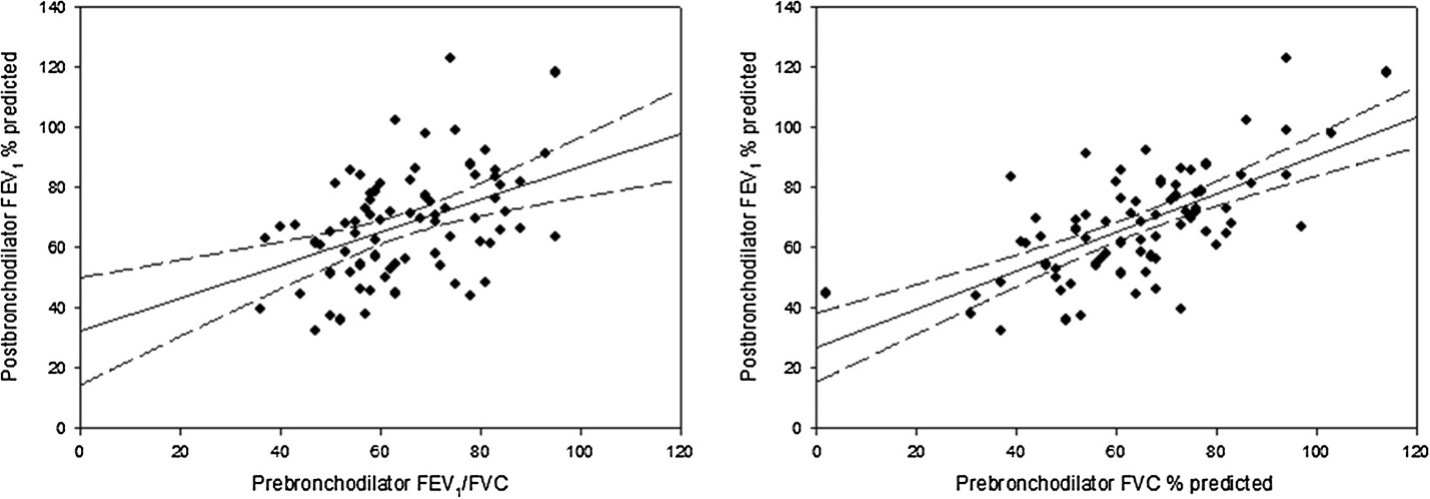
**The relationship between prebronchodilator FEV**_**1**_ **/FVC and FVC % predicted with postbronchodilator FEV**_**1**_ **% predicted.** Solid and dashed lines denote the fitted linear regression curve and 95% confidence interval, respectively.

Both prebronchodilator FEV_1_/FVC and FVC % predicted had a positive correlation with postbronchodilator FEV_1_ % predicted (Figure 4). In order to discern the relative importance of FEV_1_/FVC and FVC % predicted in contributing to the variance of postbronchodilator FEV_1_, a hierarchical multiple regression was performed. Entering FEV_1_/FVC first led to a model with R^2^ = 0.175, p < 0.001. Subsequently, entering FVC % predicted into the model led to a *change* in R^2^ of 0.508, p < 0.001, to a final R^2^ of 0.683. The combined model incorporating both FEV_1_/FVC and FVC % predicted had standardized beta coefficients of 0.517 (p < 0.001) and 0.720 (p < 0.001) respectively.

## Discussion

We aimed to elucidate the factors associated with fixed airways obstruction in a cohort of patients with severe asthma in Singapore. Smoking was found to be associated with fixed airways obstruction. Pack-year smoking history correlated modestly with the degree of fixed airways obstruction. There were also important differences in lung function in asthma patients with fixed airways obstruction. Those with fixed airways obstruction manifested with lower prebronchodilator FEV_1_ % predicted, FVC % predicted and FEV_1_/FVC. FVC was found to account for more variability than FEV_1_/FVC in predicting postbronchodilator FEV_1_ % predicted. These results suggest that both small and large airways obstruction contribute independently to fixed airways obstruction in severe asthma.

Our findings are consistent with previous reports of an association between smoking and fixed airways obstruction in severe asthmatics (the TENOR [11] and COREA [17] studies), which reported prevalences of fixed airways obstruction at 60% and 51% respectively. In both these studies, smoking was a significant factor associated with fixed airways obstruction and both studies had relatively high smoking rates among their patients (47% in TENOR and 56% in COREA). In comparison, only 18% of our cohort of 245 patients had fixed airways obstruction and our smoking rates (22%) among the Fixed Obs and Non-Fixed Obs groups are lower than the two studies, yet we found a significant association between smoking and fixed airways obstruction. We were also able to demonstrate this association despite our subjects having a lower pack-year history when compared with the COREA study. *Bumbacea et al.* [12] reported a group of patients with PostBDFEV_1_ < 50% predicted who had equivalent pack-year history to our subjects with PostBDFEV_1_ < 70% predicted, but their study was unable to demonstrate an association of fixed obstruction with smoking. Notwithstanding differences in study methodology, this discrepancy may reflect gene-environment differences, where varying thresholds of amount smoked lead to the development of fixed obstruction. Similarly, significant reductions in FVC have been reported previously in asthmatics with severe irreversible airflow obstruction [12]. However, to our knowledge, this is the first study to report that FVC is a more important predictor than FEV_1_/FVC of the degree of fixed airways obstruction.

There are several possible caveats in this study. First, there was incomplete data on bronchodilator reversibility in our cohort. This partly reflects our institutional practice of performing methacholine challenge, a test of bronchial responsiveness, as an adjunct for diagnosing asthma when patients have normal FEV_1_. Second, the subjects with irreversible obstruction may have received a misdiagnosis of asthma when in fact they have chronic obstructive pulmonary disease (COPD). However, the average age of onset of symptoms in subjects with PostBDFEV_1_ < 70% was 32.8 years (95% CI: 25.8-39.8 years), whereas the onset of symptoms in COPD occurs mostly after the age of 40 [24]. The average bronchodilator reversibility in the group with PostBDFEV_1_ < 70% was 26.8%, whereas reversibility in COPD in less marked (less than 12-15%). Furthermore, asthma was diagnosed in our patients after careful clinical evaluation by experienced clinicians in the field of allergy and respiratory diseases. Previous work has shown that in patients with similar levels of fixed obstruction, history can distinguish asthma and COPD as separate airway pathologies [25, 26]. Therefore, it is unlikely that we have inadvertently included subjects with COPD instead of asthma.

The third limitation is that the subjects in the present study who have fixed airways obstruction may belong to the asthma-COPD overlap syndrome. Overlap syndrome is recognized by coexistence of increased variability of airflow in a patient with incompletely reversible airways obstruction [27]. Patients with overlap syndrome are often excluded from clinical trials, and risk factors for the development and natural history of overlap syndrome are relatively unknown and unexplored. By definition, the difference between asthma with fixed obstruction and overlap syndrome is purely semantic and we are therefore unable to exclude that our patients have overlap syndrome. Yet, the major implication of overlap syndrome is that asthma and COPD are actually different manifestations of the same disease and share a common pathogenic origin where one condition may evolve into the other, referred to as the Dutch hypothesis [28]. The aim of the present study was not to evaluate the Dutch hypothesis, and our results do not support nor refute the Dutch hypothesis.

The most likely inference is that the subjects with PostBDFEV_1_ < 70% are asthma patients who have developed fixed obstruction in association with smoking. Our results are also in keeping with longitudinal studies showing accelerated lung function decline in asthma individuals who smoke compared to nonsmoking asthmatics [29, 30]. The reasons for this are several fold [31]: smoking reduces corticosteroid sensitivity in asthmatics [32, 33]; exposure to cigarette smoke enhances not only allergic Th2-driven inflammation [34], but also Th1-mediated (neutrophilic) inflammatory responses [33] which are not normally responsive to corticosteroids; smoking causes impaired ciliary function [35] thus renders smokers more prone to upper and lower respiratory tract infections i.e. more variability than frequent exacerbations [36].

Our results also shed light on the underlying pathophysiological derangements associated with incomplete reversibility of FEV_1_ following bronchodilator challenge in asthma. FEV_1_ as a measure of obstruction may be partitioned into components of air trapping (indicated by FVC and reflecting small airways disease) and airflow limitation (indicated by FEV_1_/FVC and reflecting large airway luminal caliber) [37–40]. Asthma has traditionally been attributed to large airway pathology [41], but we found that FVC accounted for more variability than FEV_1_/FVC in predicting postbronchodilator FEV_1_, suggesting that small airways disease is at least as important as large airways disease in contributing to fixed airways obstruction. Our results resonate with an early study [42] localizing the site of obstruction to the small airways in asthmatics with fixed obstruction by analyzing the variation in maximum expiratory flow volume curves at different gas densities. More recently, air trapping as a radiological finding has been reported as a significant determinant of fixed obstruction in asthma [39, 43]. Furthermore, air trapping and small airways disease is a unique feature of severe asthma that is absent in non-severe asthma [44]. Preliminary studies show promising results of therapies targeting distal airways, such as ultrafine bronchodilators and inhaled corticosteroids [45] and leukotriene antagonists [46], but whether these treatments can avert the development of fixed obstruction remains to be elucidated.

Our study also underscores the importance of smoking cessation in asthma patients to mitigate the development of irreversible airways obstruction. Several anti-smoking strategies have been in place in Singapore since the 1970s, including mandatory graphic health warnings on tobacco products, prohibition of tobacco advertisements and promotion, heavy tobacco taxation and a ban on smoking in public places. Smoking prevalence in Singapore has declined from 20% in 1984 to 12.6% in 2004, among the lowest rates in the world. Despite this, we are confronted with a fairly high prevalence of smoking in our cohort of severe asthmatics (22%). This implies the need for intensified efforts to promote smoking cessation in asthma patients in order to harness the beneficial effects of smoking cessation on asthma such as improvements in symptoms and lung function [47].

## Conclusions

Smoking is associated with fixed airways obstruction in patients with treatment-resistant severe asthma in Singapore. Our findings underscore the importance of smoking cessation in asthma patients to mitigate the development of irreversible airways obstruction. In addition, FVC % predicted was found to account for more variability than FEV_1_/FVC in predicting postbronchodilator FEV_1_ % predicted. These results suggest that both small and large airways disease contribute independently to fixed airways obstruction in severe asthma.

### Ethics approval

This study was approved by the Singhealth Centralised Institutional Review Board. The approval number was CIRB 2010/810/C.

## Abbreviations

SGH-SAPS: Singapore General Hospital-Severe Asthma Phenotype Study
FEV_1_: Forced expiratory volume in one second
FVC: Forced vital capacity
PostBDFEV_1_: Postbronchodilator forced expiratory volume in one second
NA: Not applicable
NS: Not significant
SD: Standard deviation.
